# 

**Published:** 1905-03

**Authors:** 


### THE BRISTOL

### Medico-Chirurgical Journal.

A JOURNAL OF THE MEDICAL SCIENCES FOR THE WEST OF ENGLAND AND SOUTH WALES

*PUBLISHED UNDER THE AUSPICES OF*

*THE BRISTOL MEDICO-CHIRURGICAL SOCIETY.*

EDITOR:

R. SHINGLETON SMITH, M.D., B.Sc.,

WITH WHOM ARE ASSOCIATED

J. MICHELL CLARKE, M.A., M.D., J. LACY FIRTH, M.S., M.D., AND JAMES SWAIN, M.S., M.D.

ASSISTANT-EDITOR:

P. WATSON WILLIAMS, M.D.

EDITORIAL SECRETARY:

JAMES TAYLOR

"Scire est nescire, nisi id me

Scire alius sciret."

LIBRARY

SURGEON GENERAL'S OFFICE

JUL. 30. 1906

192436.

VOL. XXIII.

BRISTOL: J. W. ARROWSMITH.
LONDON: J. & A. CHURCHILL, 7 GREAT MARLBOROUGH STREET.
1905.

W1
BR323

J.W. Arrowsmith

Publisher

Bristol

3375-5

### CONTENTS OF VOLUME XXIII.

| | Page |
|---|---|
| The Prevention of Apoplexy. By T. CLIFFORD ALLBUTT, M.D., F.R.S. ... ... ... ... ... ... ... ... ... | 1 |
| Some Notes on Examinations for Life Assurance. By R. SHINGLETON SMITH, M.D. Lond., F.R.C.P. ... ... ... ... ... | 11 |
| Nephropexy by Means of the Application of Strong Carbolic Acid and Temporary Support, with Report of Eight Cases. By T. CARWARDINE, M.S., M.B. Lond., F.R.C.S. ... ... ... | 24 |
| On the Advantages of Enucleation of the Tonsils over their Removal by the Guillotine. By ERNEST W. HEY GROVES, M.D., B.S., B.Sc. Lond. ... ... ... ... ... ... ... ... | 32 |
| Three Cases of Ovarian Tumour Complicating Pregnancy, Labour and the Puerperium Respectively. By EWEN J. MACLEAN, M.D., M.Ch. Edin., M.R.C.P. Lond., F.R.S. Edin. ... ... | 40 |
| On Some Symptoms of Cerebellar Tumours. By J. MICHELL CLARKE, M.A., M.D. Cantab., F.R.C.P. ... ... ... ... | 97 |
| The Causes and Treatment of Baldness. By HENRY WALDO, M.D., M.R.C.P. ... ... ... ... ... ... ... ... | 107 |
| Acne Vulgaris and its Treatment. By W. KENNETH WILLS, M.A., M.B., B.C. Camb. ... ... ... ... ... ... ... | 113 |
| The Progress of Pharmacy and Pharmaceutical Chemistry. By OLIVER C. M. DAVIS, B.Sc. Lond. ... ... ... ... ... ... | 120 |
| Cases of Joint Swellings Simulating Gout and Rheumatism. By R. LLEWELYN JONES, M.B. Lond. ... ... ... ... ... ... | 127 |
| A New Scheme for Infant Milk Depôts. By J. M. FORTESCUE-BRICKDALE, M.A., M.D. Oxon. ... ... ... ... ... | 132 |
| Sprue. By CHARLES BEGG, M.B., C.M. Edin. ... ... ... | 138 |
| Inebriety and the So-called Cures. By JAMES STEWART, B.A., F.R.C.P. Edin. ... ... ... ... ... ... ... ... ... | 144 |
| The Relation of Medicine to the Natural Sciences. By J. MICHELL CLARKE, M.A., M.D. Cantab., F.R.C.P. ... ... ... ... | 193 |

CONTENTS.

Page

The Surgical Aspect of Cholelithiasis. By JAMES SWAIN, M.S., M.D. Lond., F.R.C.S. ... ... ... ... ... ... ... 208

Twin X-ray Representation and the Reflecting Stereoscope. By WILLIAM COTTON, M.D. Edin. ... ... ... ... ... 216

A Study of the Records of One Hundred and Fifty-five Cases of Operation for Appendicitis. By CHARLES A. MORTON, F.R.C.S. 223

A Note on Congenital Dilatation of the Ureters with Hydronephrosis. By J. M. FORTESCUE-BRICKDALE, M.A., M.D. Oxon. 231

On Some Anomalous Cases of Locomotor Ataxy. By F. H. EDGEWORTH, M.B. Cantab., D.Sc. Lond. ... ... ... ... 234

The Family Doctor. By JOHN DACRE, M.R.C.S. ... ... ... 289

On Two Cases of Myasthenia Gravis. By J. MICHELL CLARKE, M.A., M.D. Cantab., F.R.C.P. ... ... ... ... ... 308

The Long Fox Lecture. By R. SHINGLETON SMITH, M.D., B.Sc. Lond., F.R.C.P. ... ... ... ... ... ... ... 317

Notes on the Voluntary Notification of Phthisis, and on the Municipal Attitude in Bristol in regard to the Disease. By D. S. DAVIES, M.D. Lond. ... ... ... ... ... ... ... 351

A Study of the Records of One Hundred and Fifty-five Cases of Operation for Appendicitis (*continued*). By CHARLES A. MORTON, F.R.C.S. ... ... ... ... ... ... ... ... ... 360

PROGRESS OF THE MEDICAL SCIENCES ... ... 49, 148, 239, 368

REVIEWS OF BOOKS ... ... ... ... ... 60, 161, 254, 375

EDITORIAL NOTES ... ... ... ... ... 73, 173, 270, 380

NOTES ON PREPARATIONS FOR THE SICK ... 76, 177, 278, 387

THE LIBRARY OF THE BRISTOL MEDICO-CHIRURGICAL SOCIETY ... ... ... ... .. ... 82, 181, 281, 392

MEETINGS OF SOCIETIES ... .. ... ... ... 84, 184, 395

OBITUARY ... ... ... ... ... ... 92, 189, 284, 398

LOCAL MEDICAL NOTES ... ... ... ... 95, 190, 285, 398

### The Library of the Bristol Medico-Chirurgical Society.

*The following donations have been received since the publication of the List in December:*

*February 28th, 1905.*

| | |
|---|---|
| L. M. Griffiths (1) ... ... ... ... ... ... ... ... | 2 volumes. |
| Laryngological Society of London (2) ... ... ... | 1 volume. |
| Medical Officer of the London County Council (3) | 1 ,, |
| Luzerne County Medical Society (4) ... ... ... | 1 ,, |
| Otological Society of the United Kingdom (5) ... | 1 ,, |
| Pathological Society of London (6)... ... ... ... | 1 ,, |
| Rhode Island Medical Society (7) ... ... ... ... | 1 ,, |
| The Scholastic Trading Company (8) ... ... ... | 1 ,, |
| Society for the Study of Disease in Children (9) ... | 1 ,, |

### FIFTY-FIFTH LIST OF BOOKS.

The titles of books mentioned in previous lists are not repeated.

The figures in brackets refer to the figures after the names of the donors, and show by whom the volumes were presented. The books to which no such figures are attached have either been bought from the Library Fund or received through the *Journal.*

| | | | |
|---|---|---|---|
| *Atlas of Illustrations of Clinical Medicine, Surgery and Pathology, An.* | Fasc. XIII. ... ... ... ... ... ... ... ... ... | | 1904 |
| **Bain, W.** [Ed.] ... | *A Text-Book of Medical Practice* ... ... ... ... ... ... | | 1904 |
| **Barwell, R.** ... | *Lateral Curvature of the Spine* ... ... ... | 6th Ed. | 1905 |
| **Burton-Fanning, F. W.** | *The Open-Air Treatment of Pulmonary Tuberculosis* | | 1905 |
| **Constant, T. E.** ... | *The Naked-Eye Anatomy of the Teeth* ... ... ... ... ... | | 1905 |
| **Cuff, Isla Stewart and H. E.** | *Practical Nursing* ... ... ... | New Ed. | 1904 |
| **Fernie, W. T.** ... | *Meals Medicinal* ... ... ... ... ... ... ... ... ... ... | | 1905 |
| **Gant, F. J.** ... | *Auto-Biography* ... ... ... ... ... ... ... ... ... ... | | 1905 |
| **Grimsdale, H. B.** | *Ophthalmic Surgery* ... ... ... ... ... ... ... ... ... | | 1904 |
| **Hirshberg, L. K.** ... | *Light as a Therapeutic Agent* ... ... ... ... ... | (7) | 1904 |
| **Latham, A.** ... ... | *Pulmonary Consumption* ... ... ... ... ... | 2nd Ed. | 1905 |
| **McKay, W. J. S.** ... | *The Preparation and After-Treatment of Section Cases* | | 1905 |
| **Macnaughton-Jones, H.** | *Manual of Diseases of Women* ... ... | 9th Ed. | 1904 |
| *Pamphlets* ... ... | 8 vols. ... ... ... ... ... ... ... ... ... ... ... ... | | [V.D.] |
| **Poincaré, L.** ... ... | *Prophylaxie et Géographie médicale* ... ... ... | (1) | 1884 |
| ,, ,, ... ... | *Traité de Hygiène Industrielle* ... ... ... ... ... | (1) | 1886 |
| **Pugh, W. T. G.** ... | *The Infectivity and Management of Scarlet Fever* ... | | 1905 |

**Reed, B.** ... ... *Diseases of the Stomach and Intestines* ... ... ... ... ... 1905
**Sequeira, J. H.** ... *Light Treatment for Nurses* ... ... ... ... ... ... ... 1905
**Shaw-Mackenzie, J. A.** *Hypodermic Medication in Inoperable Cancer* ... 1904
**Stewart and H. E. Cuff, Isla** *Practical Nursing* ... ... ... ...New Ed. 1904
**Williams, C** ... *A Short Essay on Insanity* ... ... ... ... ... ... 1905

### TRANSACTIONS, REPORTS, JOURNALS, &c.

American Journal of Obstetrics, The ... ... ... ... ... ... Vol. XLIX. 1904
American Laryngological Association, Transactions of the ... ... ... 1904
American Ophthalmological Society, Transactions of the Vol. X., Part 2 1904
American Surgical Association, Transactions of the... ... Vol. XXII. 1904
Association of American Physicians, Transactions of the Vol. XIX. 1904
British Almanac, The ... ... ... ... ... ... ... ... ... ... ... ... 1905
British Medical Journal, The ... ... ... ... ... ... ... Vol. II. for 1904
Buffalo Medical Journal ... ... ... ... ... ... ... ... Vol. XLIII. 1904
Canada Lancet, The ... ... ... ... ... ... ... ... Vol. XXXVII. 1903-04
Clinical Journal, The ... ... ... ... ... ... ... ... Vol. XXIV. 1904
Columbia University, Studies from the Department of Pathology of the College of Physicians and Surgeons ... ... ... Vol. IX. 1903-04
Écho médical du Nord, L' ... ... ... ... ... ... ... ... ... ... ... 1904
Edinburgh Medical Journal, The ... ... ... ... ... N.S., Vol. XVI. 1904
Edinburgh Obstetrical Society, Transactions of the ... ... Vol. XXIX. 1904
Hazell's Annual ... ... ... ... ... ... ... ... ... ... ... ... ... 1905
Hospital, The ... ... ... ... ... ... ... ... ... Vol. XXXVI. 1904
Hospitals—
  Guy's Hospital Reports ... ... ... ... ... ... Vol. LIX. 1905
  Saint Bartholomew's Hospital Reports ... ... ... ...Vol. XL. 1905
  Saint Thomas's Hospital Reports ... ... ... ... Vol. XXXII. 1904
Journal of Tropical Medicine, The .. ... ... ... ... Vol. I. 1898-99
Lancet, The ... ... ... ... ... ... ... ... ... ... ... Vol. II. for 1904
Laryngological Society of London, Proceedings of the ... (2) Vol. XI. 1904
Laryngoscope, The ... ... ... ... ... ... ... ... ... ... Vol. X. 1901
London County Council, Report of the Medical Officer for 1903 ... (3) 1904
Luzerne County Medical Society, Transactions of the ... (4) Vol. XII. 1904
Medical Directory, The ... ... ... ... ... ... ... ... ... ... ... 1905
Medical Record, The ... ... ... ... ... ... ... ... ... Vol. LXV. 1904
Münchener medizinische Wochenschrift ... ... .. ... Vol. II. for 1904
New Hampshire Medical Society, Transactions of the ... ... ... ... 1904
Odontological Society of Great Britain, Transactions of the, Vol. XXXVI. 1904
Ophthalmological Society of the United Kingdom, Transactions of the Vol. XXIV. 1904
Otological Society, Transactions of the ... ... ... ... (5) Vol. V. 1904
Pathological Society of London, Transactions of the ... (6) Vol. LV. 1904
Pennsylvania Medical Journal, The ... ... ... ... ... Vol. VII. 1904
Polyclinic, The ... ... ... ... ... ... ... ... ... ... Vol. VIII. 1904
Progressive Medicine ... ... ... ... ... ... ... ... ... Vol. IV. 1904
Practitioner, The ... ... ... ... ... ... ... ... [Vol. LXXIII.] 1904
Public Health ... ... ... ... ... ... ... ... ... ...Vol. XVI. 1903-04
Publishers' Circular ... ... ... ... ... ... ... ... (8) Vol. LXXX. 1904

| | | |
|---|---|---|
| Revue hebdomadaire de Laryngologie ... ... ... ... ... | Tome XXIV. | 1904 |
| St. Louis Medical and Surgical Journal | Vols. LXXXVI., LXXXVII. | 1904 |
| Sanitary Record, The ... ... ... ... ... ... ... | Vol. XXXIV. | 1904 |
| Smithsonian Institution, Annual Report for 1903 ... ... ... ... ... | | 1904 |
| Society for the Study of Disease in Children, Reports of the (9) | Vol. IV. | 1903-04 |
| Whitaker's Almanack ... ... ... ... ... ... ... ... ... ... ... | | 1905 |
| Who's Who ... ... ... ... ... ... ... ... ... ... ... ... ... ... | | 1905 |
| Year-Book of Scientific and Learned Societies ... ... ... ... ... ... | | 1904 |

### MEETINGS OF SOCIETIES.

### Bristol Medico-Chirurgical Society.

### Local Medical Notes.

**University College, Bristol.**—Students of the College have been successful in the following examinations:—

UNIVERSITY OF LONDON, M.D.—A. R. Short (Gold Medal), J. J. S. Lucas.

M.B., Intermediate Examination.—A. E. Iles. *Organic Chemistry only:* P. C. Field.

CONJOINT BOARD OF LONDON.—*Chemistry:* F. C. Morgan. *Biology:* R. S. S. Statham. *Practical Pharmacy:* P. Moxey.

*Medicine:* L. N. Morris,* F. Shingleton Smith, L. Shingleton Smith. *Midwifery:* F. G. Bergin, W. J. H. Pinniger, F. Shingleton Smith.

L.S.A.—*Medicine:* P. F. Howden.*

**Bristol Royal Infirmary.**—R. Shingleton Smith, M.D., F.R.C.P., having retired from the Honorary Visiting Staff, has been elected Consulting Physician; and P. Watson Williams, M.D., has been elected Physician to the Bristol Royal Infirmary.

**The Winsley Sanatorium.**—The rules for the admission of patients are as follows:—

I.—Only patients suffering from pulmonary and laryngeal tuberculosis in the early stages, or where a reasonable prospect is afforded of marked alleviation or cure by treatment in the Sanatorium, will be admitted.

II.—Patients will not be eligible for admission to the Sanatorium unless *bona fide* residents in one or other of the counties of Somerset, Gloucester, and Wilts.

III.—Only those persons who are not recipients of Poor Law relief, but who are unable to pay more than 15s. per week, are eligible as patients.

IV.—No patient will be admitted under 10 or over 60 years of age.

V.—Application for the admission of patients to the non-maintained beds will be considered by the Committee of Management strictly in the order in which they are received; but the Committee reserve power to decline any application if in their uncontrolled discretion they shall think fit, and they shall not be called upon to give any reason for so doing.

VI.—No patients will be admitted without certificates from his or her medical attendant, and from a member of the Medical Consultative Board of the Sanatorium, in the forms provided, together with an order from the Secretary.

VII.—Patients who have not been nominated to maintained beds will be required to pay a sum not exceeding 10s. per week, which must be paid monthly in advance, the balance of such deposit to be refunded if the patient has for any cause other than misconduct to leave the Sanatorium before the completion of the term.

VIII.—Patients will, as a rule, be admitted for a period not exceeding sixteen weeks; but this period may be curtailed by the Committee of Management if it is deemed advisable, or may be extended for a further period under special circumstances.

* Completes examination.

### Bristol Medico-Chirurgical Society.

### President.
REGINALD EAGER, M.D. Lond.

### President-Elect.

JOHN DACRE.

### Honorary Secretary.

H. F. MOLE.

### Committee.

*Past Presidents.*
L. M. GRIFFITHS.
R. SHINGLETON SMITH, M.D., B.Sc. Lond.
NELSON C. DOBSON.
F. R. CROSS, M.B. Lond.
E. MARKHAM SKERRITT, M.D., B.S., B.A. Lond.
A. J. HARRISON, M.B. Lond.
ARTHUR W. PRICHARD.
J. E. SHAW, M.B., C.M. Ed.
R. ROXBURGH, M.B. Ed.
W. H. HARSANT.
D. S. DAVIES, M.D. Lond.
BARCLAY J. BARON, M.B., C.M. Edin.
G. MUNRO SMITH.
J. PAUL BUSH, C.M.G.

J. MICHELL CLARKE, M.D., M.A. Cantab.
JOHN DACRE.
B. M. H. ROGERS, M.D., B.Ch., B.A. Oxon.
C. K. RUDGE.
JAMES TAYLOR.
H. WALDO, M.D., C.M. Aberd.
P. WATSON WILLIAMS, M.D. Lond.

Medical Practitioners wishing to join the Society are requested to communicate with the Hon. Secretary, H. F. MOLE, 1 Tottenham Place, Clifton, Bristol. The Annual Subscription is 21/-.

---

*Extracts from Journal By-laws.*

4.—The *Journal* shall be delivered free to Subscribers and also to Members of the Society whose subscriptions are not in arrear.

6.—The Annual Subscription to the *Journal* for those who pay before the 1st of July shall be 5/-, post free, or 6/- if paid after that date.

---

Communications intended for insertion in the *Journal* should be sent to the Editor, Dr. SHINGLETON SMITH, Deepholm, Clifton Park, Clifton, Bristol.

Books for Review and Exchange Journals should be sent to the Assistant-Editor, Dr. P. WATSON WILLIAMS, Medical Department, University College, Bristol.

Communications referring to the delivery of the *Journal* should be sent to the Editorial Secretary, Mr. JAMES TAYLOR, 36 Alma Road, Clifton, Bristol, to whom Subscriptions are to be paid *in advance*.

Cases for Binding Vols. I.–XXII. are now ready, and may be obtained, price 7*d*. each (postage 2*d*. extra), upon application to J. W. ARROWSMITH, Quay Street, Bristol; or the *Journal* will be bound, including Case, 1/3 per volume.

### Bristol Medico-Chirurgical Society.

---

### PAST PRESIDENTS.

1874—75. FREDERICK BRITTAN, M.D., B.A. Dub.
1875—76. ROBERT WILLIAM COE.
1876—77. SAMUEL MARTYN, M.D. Ed.
1877—78. AUGUSTIN PRICHARD, M.D. Berlin.
1878—79. HENRY EDWARD FRIPP, M.D. Würzburg.
1879—80. WILLIAM MICHELL CLARKE.
1880—81. JOSEPH GRIFFITHS SWAYNE, M.D. Lond.
1881—82. EDWARD LONG FOX, M.D. Oxon.
1882—83. JAMES GEORGE DAVEY, M.D. St. And.
1883—84. WILLIAM JOHNSTONE FYFFE, M.D., B.A. Dub.
1884—85. GEORGE FORSTER BURDER, M.D. Aberd.
1885—86. WILLIAM HENRY SPENCER, M.D., M.A. Cantab.
1886—87. FRANCIS POOLE LANSDOWN.
1887—88. LEMUEL MATTHEWS GRIFFITHS.
1888—89. ROBERT SHINGLETON SMITH, M.D., B.Sc. Lond.
1889—90. NELSON CONGREVE DOBSON.
1890—91. SAMUEL HENRY SWAYNE.
1891—92. FRANCIS RICHARDSON CROSS, M.B. Lond.
1892—93. EDWARD MARKHAM SKERRITT, M.D., B.S., B.A. Lond.
1893—94. JAMES GREIG SMITH, M.B., C.M., M.A. Aberd., F.R.S.E.
1894—95. ALFRED JAMES HARRISON, M.B. Lond.
1895—96. ARTHUR WILLIAM PRICHARD.
1896—97. ALFRED EDWARD AUST LAWRENCE, M.D., C.M. Aberd.
1897—98. JOHN EDWARD SHAW, M.B., C.M. Ed.
898—99. ROBERT ROXBURGH, M.B. Ed.
1899—00. WILLIAM HENRY HARSANT.
1900—01. DAVID SAMUEL DAVIES, M.D. Lond.
1901—02. BARCLAY JOSIAH BARON, M.B., C.M. Ed.
1902—03. GEORGE MUNRO SMITH.
1903—04. JAMES PAUL BUSH, C.M.G.

1905.

### LIST OF SUBSCRIBERS

TO THE

### Bristol Medico-Chirurgical Journal.

*Those marked * are Members of the Society.*

| | |
|---|---|
| Ackland, C. H. | Moorland Court, Poole Road, Bournemouth. |
| Ackland, J. McKno | 24 Southernhay, Exeter. |
| *Ackland, W. R. | 5 Rodney Place, Clifton. |
| Adye, W. J. A. | Church House, Bradford, Wilts. |
| Aldous, George F. | Charlton House, Mannamead, Plymouth. |
| Aldridge, Charles, M.D., C.M. Aberd. | Plympton. |
| *Alexander, D.A., M.B., Ch.B. Vict. | 30 Berkeley Square, Clifton. |
| Alexander, James, M.D., M.Ch., B.A. Dub. | Bishop's Place, Paignton. |
| Ambrose, J., M.B., C.M. Aberd. | 138 Fishponds Road, Eastville, Bristol. |
| Aveline, H. T. S. | Somerset Asylum, Taunton. |

| | |
|---|---|
| *Ballance, H. Stanley, M.D. Lond. | 4 Royal Terrace, Weston-super-Mare. |
| Barber, M. C. | Glenavon House, Staple Hill, Bristol. |
| Baretti, T. G. L'Enardi | 49 Royal York Crescent, Clifton. |
| *Barker, G. H., M.B., B.Sc. Lond. | 124 Redland Road, Bristol |
| *Baron, Barclay J., M.B., C.M. Ed. | 16 Whiteladies Road, Clifton. |
| *Basset, Walter | 24 Stow Hill, Newport, Mon. |
| Batten, Rayner W., M.D. Lond. | 1 Brunswick Square, Gloucester. |
| Batten, W. Smith | West Hill, Calne. |
| *Beddoe, John, M.D. Ed., B.A. Lond., F.R.S. | The Chantry, Bradford-on-Avon. |

### LIST OF SUBSCRIBERS.

| | |
|---|---|
| BENNETT, W. S. | Escot, Penzance. |
| BERNARD, C. | 2 Spencer Terrace, Fishponds. |
| *BERRY, F. C., M.D., B.Ch., B.A. Dub. | Burnham, Somersetshire. |
| BERRY, JAMES, M.B., B.S. Lond. | 21 Wimpole Street, London, W. |
| *BEVERLEY, K. H. | Children's Hospital, Bristol. |
| BIAMONTI, SEBASTIANO | 27 Via Balbi, Genoa, Italy. |
| *BLACKER, A. E. | 20 Victoria Square, Clifton. |
| *BLAGG, ARTHUR F., M.D. Durh. | 28 Caledonia Place, Clifton. |
| BOYCE, JAMES G. | The Chipping, Wotton-under-Edge. |
| BOYD, GEO. S. J. | 19 St. George's Square, Stamford, Lincs. |
| *BRASHER, CHARLES W. J. | 128 Cotham Brow, Bristol. |
| *BRISTOWE, H. C., M.D. Lond. | Wrington, Somerset. |
| BROWN, G. ARTHUR | Tredegar. |
| BROWN, WILLIAM, M.D., C.M. Glasg. | Park View, Fishponds, Gloucestershire. |
| *BULLEN, F. ST. JOHN | 12 Pembroke Road, Clifton. |
| *BUNBURY, E. G. | 13 Dowry Square, Hotwells. |
| BURROUGHS, E. F. H. | 3 Elgin Park, Bristol. |
| *BUSH, J. PAUL, C.M.G. | Vyvyan House, Clifton Park, Clifton. |

| | |
|---|---|
| *CAMPBELL-HORSFALL, C. E., M.B., Ch.B. (Vict.) | Sisson Lodge, Clevedon. |
| *CARTER, T. M. | 28 Victoria Square, Clifton. |
| *CARWARDINE, THOMAS, M.B., M.S. Lond. | 16 Victoria Square, Clifton. |
| *CAVE, EDWARD J., M.D. Lond. | 20 Circus, Bath. |
| CHUNG, W. C. | Imperial Chinese Hospital, Chinanfu, Shan-tung, China. |
| CHUTE, HENRY M. | King William's Town, S. Africa. |
| *CLARKE, JOHN MICHELL, M.D., M.A. Cantab. | 28 Pembroke Road, Clifton. |
| COBBLEDICK, A. S., M.D., B.S. Lond. | 402 Brixton Road, London, S.W. |
| COCHRAN, ALEXANDER, M.B., C.M. Glasg. | Bath Road, New Brislington, Bristol. |
| *COLLINSON, T. A. | 1 Winsley Road, Cheltenham Road, Bristol. |
| *COOK, HENRI, M.D., C.M. Aberd. | 1 Dean Lane, Southville, Bristol. |
| *COOMBS, CAREY, M.D. Lond. | 11 Henleaze Road, Westbury-on-Trym. |
| *CORFIELD, C. | 70 York Road, New Cut, Bristol. |
| CORNALL, Miss | 20 Arley Hill, Bristol. |
| *CORRIGAN, W. J. | Cloughmore, Splott Avenue, Cardiff. |
| COSSHAM, W. R., M.D., C.M. Aberd. | Cirencester. |
| COTTON, WILLIAM, M.B., C.M., M.A. Ed. | 231 Gloucester Road, Bristol. |
| COULTER, R. J., M.B. Dub. | 11 Clytha Park Road, Newport, Mon. |
| COUSINS, J. WARD, M.D. Lond. | Kent Road, Southsea. |
| COWAN, F. S. | 27 Queen Square, Bath. |
| CRADDOCK, SAMUEL | South Lyncombe, Bath. |
| CRIDLAND, A. B. | 11 Waterloo Road, Wolverhampton. |
| CRISP, J. ELLIS | Alexander House, Corsham. |
| *CROSS, F. RICHARDSON, M.B. Lond. | Worcester House, Clifton. |
| *CROSSMAN, F. W. | Hambrook, Gloucestershire. |
| *CROUCH, C. PERCIVAL, M.B. Lond. | 1 Royal Terrace, Weston-super-Mare. |
| CULROSS, JAMES, M.B., C.M., M.A. Glasg. | 66 Queen Street, Newton Abbot. |

| | |
|---|---|
| *DACRE, JOHN | 14 Eaton Crescent, Clifton. |
| DALBY, AUGUSTUS W. | Argyll House, Frome. |
| *DAVIES, D. S., M.D. Lond., D.P.H. Cantab. | 60 Oakfield Road, Clifton. |
| DAVIS, THEODORE, M.D. Lond. | Beachcroft, Clevedon. |

| | |
|---|---|
| DAVY, HENRY, M.D. Lond ... ... ... ... ... | Southernhay House, Exeter. |
| DENING, EDWIN ... ... ... ... ... ... ... ... | Manor House, Stow-on-the-Wold. |
| DEVINE, H. ... ... ... ... ... ... ... ... ... | West Riding Asylum, Wakefield. |
| DICKIE, JAMES STEEL, M.D., C.M. Aberd. ... | Boscombe Park, Bournemouth. |
| *DOBSON, NELSON C. ... ... ... ... ... ... ... | Avonmore, Sneyd Park. |
| DOWN, A. R. ... ... ... ... ... ... ... ... ... | Bampton, Devon. |
| DUNBAR, ELIZA L. WALKER, M.D. Zürich ... | 9 Oakfield Road, Clifton. |
| DYMOKE, F. ... ... ... ... ... ... ... ... ... | 21 Cotham Road, Bristol. |

| | |
|---|---|
| EADON, W. F. BAILEY ... ... ... ... ... ... | Hambrook, Gloucestershire. |
| *EAGER, REGINALD, M.D. Lond. ... ... ... ... | Northwoods, Winterbourne. |
| *EAGER, W. R. ... ... ... ... ... ... ... ... ... | Northwoods, Winterbourne. |
| EBERLE, EMILY, M.A., M.B., B.Ch. Dub. ... | 17 Oakfield Road, Clifton. |
| *EDGEWORTH, F. H., M.B., B.C., B.A. Cantab., D.Sc. Lond. ... ... ... ... ... ... ... ... | 35 Pembroke Road, Clifton. |
| ELDER, WILLIAM ... ... ... ... ... ... ... ... | 4 Trelawney Road, Bristol. |
| ELLIOT BROS. LTD. ... ... ... ... ... ... ... | *Australian Pharmaceutical Notes & News*, O'Connell Street, Sydney, N.S.W. |
| *ELLIOTT, CHRISTOPHER, M.D., B.A. Dub. ... | 3 Beaufort Road, Clifton. |
| ELLIOTT, F. PERCY, M.B., C.M. Aberd. ... ... | 113 Grove Road, Walthamstow, London N.E. |
| ELLIS, W. McD., M.D. Brux. ... ... ... ... ... | 25 The Circus, Bath. |
| EVANS, T. G. C. ... ... ... ... ... ... ... ... | Budleigh Salterton, Devon. |
| EVERY-CLAYTON, L. E. V., M.D. Lond. ... ... | Rosslyn, Clevedon, Som. |

| | |
|---|---|
| FAIRBANKS, W., M.D., C.M. Ed. ... ... ... ... | Wells, Somersetshire. |
| *FERGUSON, R. S., B.A. Durh., M.B. C.M. Edin. | Elm Grove, Calne, Wilts. |
| *FIRTH, J. L., M.D., M.S. Lond. ... ... ... ... | 20 Richmond Park Road, Clifton. |
| *FISHER, THEODORE, M.D. Lond.... ... ... ... | Harley Lodge, Clifton. |
| *FLEMMING, A. L. ... ... ... ... ... ... ... ... | Richmond Park Road, Clifton. |
| *FLEMMING, C. E. S. ... ... ... ... ... ... ... | Manvers House, Bradford-on-Avon. |
| *FLETCHER, J., M.D., C.M., D.P.H. Aberd. ... | Ham Green, Somerset. |
| FLOOD, P. ... ... ... ... ... ... ... ... ... ... | 318 Stapleton Road, Bristol. |
| *FORTESCUE-BRICKDALE, J. M., M.A., M.D., B.Ch. Oxon.... ... ... ... ... ... ... ... ... | 52 Pembroke Road, Clifton. |
| FORTY, D. H. ... ... ... ... ... ... ... ... ... | Wotton-under-Edge. |
| FOSS, E. V. ... ... ... ... ... ... ... ... ... | Babbacombe House, Ashton Road, Bedminster, Bristol. |
| FOWLER, O. H. ... ... ... ... ... ... ... ... ... | Cirencester. |
| FOX, F. F. ... ... ... ... ... ... ... ... ... ... | Yate House, Chipping Sodbury. |
| FOX, T. COLCOTT, M.B. Lond., B.A. Cantab.... | 14 Harley Street, London, W. |
| *FRASER, FORBES ... ... ... ... ... ... ... ... | 2 The Circus, Bath. |
| FRAZER-TOOVEY, T. E. ... ... ... ... ... ... ... | Balfour House, Downs Road, Clapton, London, N.E. |
| FREEMAN, J., M.D. Brux. ... ... ... ... ... ... | Glencairn Villa, Queen's Road, Clifton. |

| | |
|---|---|
| GARLAND, E. B., M.B., C.M. Edin. ... ... ... | 92 Hampton Road, Redland. |
| GENGE, T. T. ... ... ... ... ... ... ... ... ... | 21 Whiteladies Road, Bristol. |
| *GERRISH, D. S. ... ... ... ... ... ... ... ... | 322 Church Road, St. George, Bristol. |
| *GIBBS, ALFRED N. GODBY ... ... ... ... ... | 52 Whiteladies Road, Clifton. |
| GIDLEY, G. G. ... ... ... ... ... ... ... ... ... | Cullompton, Devon. |
| GOSS, T. BIDDULPH... ... ... ... ... ... ... ... | 1 The Circus, Bath. |

### LIST OF SUBSCRIBERS.

Grace, Alfred ... ... ... ... ... ... ... Chipping Sodbury.

Grace, Edward Mills... ... ... ... ... ... Thornbury, Gloucestershire

Gratte, C. Brooke ... ... ... ... ... ... 183 Commercial Road, Newport, Mon.

*Green, T. A., M.D., C.M. Edin. ... ... ... 33*a* Whiteladies Road, Clifton.

*Greer, W. J. ... ... ... ... ... ... ... ... 2 Chepstow Road, Newport, Mon.

*Griffiths, Lt.-Col. G.S. ... ... ... ... ... 11 Vyvyan Terrace, Clifton.

*Griffiths, J. S. ... ... ... ... ... ... ... 20 Redland Park, Bristol.

*Griffiths, L. M. ... ... ... ... ... ... ... 11 Pembroke Road, Clifton.

*Groves, E. W. H., M.B., B.Sc. Lond. ... ... 16 Richmond Hill, Clifton.

Hadwen, W. R., M.D. St. And.... ... ... ... 35 Brunswick Square, Gloucester.

*Hailes, Clements D. G., M.D., C.M. Ed. ... 27 Alma Road, Clifton.

*Haines, A. H. ... ... ... ... ... ... ... ... 8 Cotham Road, Bristol.

*Hall, E. G., M.B. Lond. ... ... ... ... ... 53 Hampton Road, Redland, Bristol.

Handfield-Jones, Montagu, M.D. Lond. ... 35 Cavendish Square, London, W.

*Harris, H. Elwin, B.A., M.B. Cantab. ... ... 13 Lansdown Place, Clifton.

*Harrison, A. J., M.B. Lond. ... ... ... ... Guthrie Road, Clifton.

*Harsant, W. H. ... ... ... ... ... ... ... Tower House, Clifton.

*Harty, Garnett, M.B., Ch.B. Edin. ... ... Eye Hospital, Bristol.

*Harvey, Alfred, M.B. Lond. ... ... ... ... Westbury-on-Trym.

Hay, M. B.... ... ... ... ... ... ... ... ... The Avenue, Surbiton, Surrey.

Hayman, Alfred G. ... ... ... ... ... ... Hapsford House, near Frome.

*Hayman, C. A., M.D. St. And. ... ... ... ... Kingston Villa, Richmond Hill, Clifton.

Heath, Miss ... ... ... ... ... ... ... ... Isolation Hospital, Taunton.

*Heaven, John C., D.P.H. Lond. ... ... ... 17 Whiteladies Road, Bristol.

*Herapath, C. K. C. ... ... ... ... ... ... Cotham Park, Bristol.

*Hetling, H. E. ... ... ... ... ... ... ... 30 Elliston Road, Bristol.

Hill, Hedley ... ... ... ... ... ... ... ... 1 Redcliff Hill, Bristol.

*Hill, Walter J. ... ... ... ... ... ... ... The Brow, Clevedon.

Hoffman, A. W. W. ... ... ... ... ... ... 30 The Parade, Leamington.

*Holmes, Thomas E., M.A., M.B., B.C. Cantab. Rosendal, Redland Road, Bristol.

Hospital, Bristol General (*Sec.*, W. Thwaites).

Hospital, Taunton and Somerset (*House-Surgeon*, W. Drake).

Huggard W. R., M.D., M.Ch., M.A. Roy. Univ. ... ... ... ... ... ... ... ... ... Davos-Platz, Switzerland.

Imlay, G. A., M.B., C.M. Aberd. ... ... ... 1 Cotham Park, Bristol.
Ingle, C. Durban ... ... ... ... ... ... ... Somerton, Somerset.

Jamison, A., M.B., B.Ch., M.A. Roy. Univ. I. 66 Woodstock Road, Belfast.
Jones, Evan ... ... ... ... ... ... ... ... Ty-mawr, Aberdare.
*Jones, E. J. Trevor, M.D. Brux. ... ... ... Aberdare, Glamorganshire.
Jones, H. Macnaughton, M.D., M.Ch. Roy. U.I.... ... ... ... ... ... ... ... ... 131 Harley Street, London, W.

| | |
|---|---|
| *Kemm, F. St. J. | Worle. |
| *Kendall, H. W. | 1 Burlington Road, Redland. |
| *Kyle, H. G., M.B., B.Ch. Oxon. | 31 Westbury Road, Westbury-on-Trym. |

| | |
|---|---|
| *Lace, F. | 1 Paragon, Bath. |
| Lancaster, E. Le Cronier, B.A., M.B., B.Ch. Oxon. | Winchester House, Swansea. |
| *Lansdown, R. G. P., M.B., B.S. Dur. | 39 Oakfield Road, Clifton. |
| Lawrie, J. Macpherson, M.D., C.M. Glasg. | Greenhill, Weymouth. |
| Le Soudier, H. | 174 et 176 Boulevard St. Germain, Paris |
| *Lees, E. Leonard, M.D., C.M. Ed. | 1 Chesterfield Place, Clifton. |
| Leigh, W. W. | Treharris, Glamorganshire. |
| *Leonard, R. C., M.D. Ed. | 134 Coronation Road, Bristol. |
| *Ligertwood, Walter | Bath and Somerset County Asylum, Wells. |
| Lister, Lord, P.R.S. | 12 Park Crescent, London, W. |
| Lowe, T. Pagan | 16 Circus, Bath. |
| *Lucas, J. J. S., M.B. Lond. | 186 Wells Road, Totterdown. |
| Lucy, Reginald H. | 9 The Crescent, Plymouth. |

| | |
|---|---|
| MacCarthy, Ffennell, M.D. Dub. | 21 Alexandra Road, Westbury Park. |
| *McCrea, P. W., M.D., B.Ch., B.A.O., B.A. Dub. | 19 Portland Square, Bristol. |
| MacDonald, P. W., M.D., C.M. Aberd. | Herrison, Dorchester. |
| *McClelland, A. W., M.B., C.M. Glasg. | Clarendon House, Wick Road, Brislington. |
| *McKee, A. B., M.B., B.Ch. Dub. | Sussex Villa, Sussex Place, Bristol. |
| Mackinnon, Charles, M.B., C.M., M.A. Glasg. | Davaar, Cirencester. |
| Maclean, Ewen J., M.D., C.M. Ed. | 12 Park Place, Cardiff. |
| Maclean, J. Campbell, M.B., C.M. Ed. | Swindon House, Swindon, Wiltshire. |
| Macready, J. | 42 Devonshire Street, Portland Place, London, W. |
| McWatters, J. C. | Almondsbury. |
| Marsh, O. E. Bulwer | Parkdale, Newport, Mon. |
| Marshall, Arthur L., M.B., B.A. Cantab | Thornleigh, Upper Clapton, London, N.E. |
| *Martin, E. F., M.B. Ed. | 7 Royal Terrace, Weston-super-Mare. |
| *Martin, R. C. | 3 Clifton Vale, Clifton. |
| *Martyn, G. J. King, M.D., B.C., B.A. Cantab. | 12 Gay Street, Bath. |
| Mason, S. B. | 12 Park Terrace, Pontypool. |
| Mason, William | Sedgemoor Cottage, St. Austell, Cornwall. |
| Matthews, Charles E., M.D. Dur. | 6 Hickman Road, Penarth. |
| Matthews, H. E. | The Mall Pharmacy, Clifton. |
| *Melsome, W. S., M.D. Cantab. | 29 Circus, Bath. |
| Meredith, John, M.D. Ed. | Wellington, Somersetshire. |

### LIST OF SUBSCRIBERS.

| | |
|---|---|
| *Millard, W. J. Kelson, M.D. St. And. | 7 Bayshill Villas, Cheltenham. |
| Mitchell, Trafford, M.D. | Gorseinon, R.S.O., Glamorganshire. |
| *Mole, Harold F. | 1 Tottenham Place, Clifton, Bristol. |
| *Monckton, H. H. | 3 Richmond Place, Kingswood, Bristol. |
| Monckton, William | Portishead. |
| *Moore, L. A. | 29 Arley Hill, Bristol. |
| Moores, De la Hey | 6 West Mall, Clifton. |
| Morgan, Albert T., M.D. Brux. | 89 Chesterfield Road, St. Andrew's Park, Bristol. |
| *Morgan, T. Whitworth S. | The Laurels, Timsbury, Bath. |
| *Morton, C. A. | 14 Vyvyan Terrace, Clifton. |
| *Morton, W. B., M.D. Lond. | Brislington House, Brislington. |
| Munden, Charles | Ilminster. |
| *Myles, G T. | 147 Whiteladies Road, Bristol. |

| | |
|---|---|
| *Neild, Newman, M.B., B.Ch. Vict. | 9 Richmond Hill, Clifton. |
| *Nevill, W. N., M.D., B.Ch., B.A. Dub. | Leighton Villa, Southville, Bristol. |
| *Newman, Charles | 156 Cheltenham Road, Bristol. |
| *Newnham, W. H. C., M.B., M.A. Cantab. | Chandos Villa, Clifton. |
| Nicholson, T. D., M.D. Ed. | 2 Whiteladies Road, Clifton. |
| *Nixon, J. A., B.A., M.B., B.C. Cantab. | Bristol Royal Infirmary. |
| Norman, J. H. | Goodleigh, Winkleigh, Devonshire. |

| | |
|---|---|
| O'Brien, Cornelius | Devon House, Whitehall, Bristol. |
| Odell, William, M.D. Durh. | Ferndale, Torquay. |
| *Ogilvy, Alexander, M.D., B.Ch., B.A. Dub. | Montrose House, Queen's Road, Clifton. |
| Openshaw, E. H. | Cheddar. |
| *Ormerod, H. Lawrence, M.B., B.Ch., B.A.O., R.U.I. | Burnley, Westbury-on-Trym |

| | |
|---|---|
| Page, G. Shepley | 78 Old Market Street, Bristol. |
| Papillon, J. W. | Brent Knoll, Bridgwater. |
| *Parker, George, M.D., M.A. Cantab. | 14 Pembroke Road, Clifton. |
| Parry, J. H. | Ashley Road, Bristol. |
| Parsons, Francis J. C. | Ivy House, Bridgwater. |
| *Parsons, H. F. | The Heath, Stoke Bishop. |
| Parsons, J. H., M.B., B.S., D.Sc. Lond. | 27 Wimpole Street, London, W. |
| Paterson, Donald Rose, M.D., C.M. Ed. | 18 Windsor Place, Cardiff. |
| Peake, Arthur W. | 34 Gloucester Road, Bishopston, Bristol. |
| Peake, G. Arthur | Rodney Place, Cheltenham. |
| Pearse, E. M. | 2 Varley Terrace, Liskeard. |
| Peck, Lt.-Col. F.S., I.M.S. | 6 Harington Street, Calcutta. |
| Phillips, C. M., M.D. Brux. | Ravenslea, Kensington Hill, Brislington. |
| Phillips, Edward Picton | High Street, Haverfordwest. |
| *Pickering, C. F. | 13 Berkeley Square, Bristol. |
| Powell, J. J., M.D. Lond. | 2 Gloucester Road, Bishopston, Bristol. |

| | |
|---|---|
| *POWELL, L. W. | Two Mile Hill Road, Kingswood, Bristol. |
| POWELL, WILLIAM, M.B. Lond. | Hill Garden, Torquay. |
| PRICE, D. T., M.B. Lond. | Castle Cary, Somerset. |
| *PRICHARD, ARTHUR W. | 6 Rodney Place, Clifton. |
| *PROWSE, ARTHUR B., M.D. Lond. | 5 Lansdown Place, Clifton. |
| PROWSE, W. B. | 11 St. George's Place, Brighton. |
| PRUEN, S. T., M.D. Dur. | Sherborne Lodge, Cheltenham. |
| PURDY, J. R., M.D. Aber. | Lower Hutt, Wellington, New Zealand. |

| | |
|---|---|
| RANDOLPH, CHARLES | St. Michael's, Milverton, Somersetshire. |
| *RATTRAY, J. M., M.D., C.M., M.A. Aberd. | Frome. |
| *RAYNER, D. C. | 9 Lansdown Place, Clifton, Bristol. |
| REDWOOD, T. HALL, M.D., C.M., M.A. Dur. | The Lawn, Rhymney, Monmouthshire. |
| RENDALL, JOHN | Forestside, Lymington. |
| *REW, G. R. | Children's Hospital, Bristol. |
| RICHARDS, D. T. | The Meadows, Risca, Mon. |
| *RICHARDSON, A. W., M.B. Lond. | Bristol General Hospital. |
| RIDDELL, ANDREW W. | 91 Rowson Street, New Brighton, Cheshire. |
| ROBERTS, FREDERICK T., M.D., B.Sc. Lond. | 102 Harley Street, London, W. |
| RODGERS, JOHN W., M.B., C.M. Ed. | Redfield, Gloucestershire. |
| *ROGERS, B. M. H., M.D., B.Ch., B.A. Oxon. | 1 Victoria Square, Clifton. |
| *ROLFE, R., B.A., M.B., B.C. Camb. | Roseneath, Avonmouth. |
| *ROSE, F. H. | 13 Westfield Park, Clifton. |
| *ROSSITER, GEORGE F., M.B. Lond. | Cairo Lodge, Weston-super-Mare. |
| *ROUÉ, W. BARRETT, M.D., M.S. Dur. | 2 Buckingham Place, Clifton. |
| *ROXBURGH, ROBERT, M.B. Ed. | 1 Victoria Buildings, Weston-super-Mare. |
| *RUDGE, C. KING | 145 Whiteladies Road, Bristol. |
| RUFFER, M. ARMAND, M.D., B.S., M.B. Oxon | Ramleh, Alexandria, Egypt. |
| RUTHERFOORD, H. T., M.A., M.D. Camb. | Salisbury House, Taunton. |

| | |
|---|---|
| *SCOTT, EDWARD, M.D. Durh. | 25 York Crescent Road, Clifton. |
| SCOTT, RICHARD J. H. | 28 Circus, Bath. |
| SEARLE, RICHARD BURFORD | Bexley, Dartmouth. |
| SEWARD, W. J., M.B. Lond. | Asylum, Colney Hatch, London, N. |
| *SHAW, J. E., M.B., C.M. Ed. | 23 Caledonia Place, Clifton. |
| SHAW-MACKENZIE, JOHN A., M.D. Lond. | 42 Green Street, Park Lane, London, W. |
| *SHEPPARD, E. J. | 8 Richmond Hill, Clifton. |
| *SHOOLBRED, W. A. | St. Ann's, Chepstow. |
| *SHORT, A. R., M.D. Lond. | Bristol General Hospital. |
| *SIEVEKING, A. R. | Nairobi, Howard Road, Westbury Park. |
| SIMONS, R. J. | Cae Court, Bridgend. |
| SKELTON, HENRY, M.D. St. And. | Downend, Gloucestershire. |
| *SKERRITT, E. MARKHAM, M.D., B.S., B.A. Lond. | Ivor House, Durdham Park, Bristol. |
| *SMITH, G. COCKBURN, M.D. Brux. | 14 South Road, Newton Abbot. |
| *SMITH, G. MUNRO | 18 Apsley Road, Clifton. |
| SMITH, JAMES F. | Les Pieux, Cobo, Guernsey. |
| SMITH, REV. REGINALD, M.A. Cantab. | The Vicarage, Walpole St. Andrew, Norfolk. |
| *SMITH, R. SHINGLETON, M.D., B.Sc. Lond. | Clifton Park, Clifton. |
| SMITH, W., M.B. Lond. | Brabourne, Ashford, Kent. |
| SMITH, W. ALEXANDER, M.B., M.A. Oxon. | Newport, Essex. |
| *SMITH, W. A. L., M.A., M.B., B.C. Cantab. | 8 Chamberlain Street, Wells. |

### LIST OF SUBSCRIBERS.

| | |
|---|---|
| Smyth, H. J. | New Road, South Molton. |
| Society, Cardiff Medical (*Hon. Lib.*; Queen Street, Cardiff). | |
| Society, Manchester Medical | Medical Society's Library, The Owens College, Manchester. |
| Society, Plymouth Medical (*Hon. Lib.*, J. Elliott Square). | |
| *Stack, E. H. E., B.A., M.B., B.C. Cantab. | Royal Infirmary, Bristol. |
| Stamp, W. D. | Ridgeway House, Plympton. |
| Staple, J. D. | Clevedon Villa, Cheltenham Road, Bristol. |
| Statham, R. Whiteside | Cheddar, Somersetshire. |
| *Steele, Charles, M.D. Dur. | 17 Richmond Hill, Clifton. |
| *Steele, W. H., Lt.-Col., M.D. Dub. | 18 Mortimer Road, Clifton. |
| Stevens, W. H. | 26 Zetland Road, Bristol. |
| *Stewart, J., B.A., R.U.I. | Dunmurry, Sneyd Park, Bristol. |
| Stock, P. G., R.A.M.C. | c/o P.M.O., South Africa. |
| *Stock, W. S. V., M.B., B.S. Lond. | Bristol General Hospital. |
| Stoddart, F. Wallis | Southey House, College Green, Bristol. |
| *Style, H., M.A., M.B., B.C. Cantab. | Avonmouth. |
| *Swain, James, M.D., M.S. Lond. | 4 Victoria Square, Clifton. |
| *Swayne, W. Carless, M.D. Lond. | St. Paul's Road, Clifton. |
| *Symes, J. O., M.D. Lond. | 11 Richmond Hill, Clifton. |
| Symonds, H. P. | 35 Beaumont Street, Oxford. |
| Symons, John | Buriton House, Penzance. |

| | |
|---|---|
| Taylor, C. Bell, M.D. Edin. | 9 Park Row, Nottingham. |
| *Taylor, C. J. | 54 Apsley Road, Clifton. |
| *Taylor, James | 36 Alma Road, Clifton. |
| *Temple, G. H., M.B., C.M. Ed. | Weston-super-Mare. |
| Thomas, A. Garrod, M.D., C.M. Ed. | Clytha Park, Newport, Mon. |
| Thomas, J. Lynn | 21 Windsor Place, Cardiff. |
| *Thomas, J. M. M. | 5 Colston Parade, Redcliffe, Bristol. |
| Thomas, Raglan, M.D. Lond. | 13 West Southernhay, Exeter. |
| Thomson, Eustace B., M.D., C.M. Glasg. | Albany Place, Plymouth. |
| *Tivy, W. J. | 8 Lansdown Place, Clifton. |
| Tosswill, Louis H., M.B., B.A. Cantab. | 1 West Southernhay, Exeter. |
| Trevithick, Edgar, M.A., M.D. Cantab. | 24 Promenade, Cheltenham. |
| Tyley, R. P., M.D. St. And. | Wedmore. |

| | |
|---|---|
| Vachell, Herbert R., M.D., C.M. Aberd. | 18 Newport Road, Cardiff. |
| Vereker, R. H. | Eastleigh, Curry Rivel, Somerset. |

| | |
|---|---|
| *Waldo, Henry, M.D., C.M. Aberd. | 19 Pembroke Road, Clifton. |
| *Walker, Cyril H., M.B., B.A. Cantab. | 16 Pembroke Road, Clifton. |
| Wallace, Rev. Canon, M.A. Oxon. | 3 Gloucester Row, Clifton. |
| Wallace, J. T., M.B., B.Ch. R.U.I. | 48 Coronation Road, Bristol. |
| *Wallace, John | 1 Oriel Terrace, Weston-super-Mare. |
| Walsh, Leslie H., M.B., B.S. Durh. | 21 Gay Street, Bath. |
| *Walters, C. F. | 129 Redland Road, Bristol. |
| Ward, J. L. W. | Victoria Street, Merthyr Tydvil. |
| *Wathen, J. Hancocke | 16 York Place, Clifton |

LIST OF SUBSCRIBERS.

| | |
|---|---|
| WEATHERLY, LIONEL A., M.D., C.M. Aberd. | Bailbrook House, Bath. |
| WEBBER, WILLIAM W. | Crewkerne. |
| *WESTCOTT, W. G. | H.M. Dockyard, Malta. |
| *WHICHER, A. H. | 115 Queen's Road, Clifton. |
| WHITE, C. R. | Nixon Villa, Merthyr Vale, Glamorgan. |
| WHITE, J. W. | Orchard House, Nailsea. |
| *WHITE, W. PERCY | College Green, Bristol. |
| *WHITMORE, F. C. | 497 Fishponds Road, Bristol. |
| WICKHAM, W. | Hill House, Tetbury. |
| WIGAN, C. A., M.D. Dur. | Portishead. |
| WIGMORE, J., M.D. Roy. Univ. I. | 20 Green Park, Bath. |
| WILCOX, HENRY, M.B. Aberd. | Fleet, Hampshire. |
| WILLCOX, R. LEWIS | Warminster. |
| WILLETT, GEORGE G. D. | Keynsham, Somersetshire. |
| *WILLIAMS, E. C., M.B., B.C., B.A. Cantab. | 9 Whatley Road, Clifton. |
| WILLIAMS, H. EGERTON | The Mount, Newport, Mon. |
| *WILLIAMS, LIONEL H. | Thornbury, Gloucestershire. |
| *WILLIAMS, P. WATSON, M.D. Lond. | 4 Clifton Park, Clifton. |
| *WILLIAMS, W. ROGER | Beaufort House, Clifton Down, Clifton. |
| WILLIS, W. M. | 7 Regent Street, Nottingham. |
| *WILLS, W. K., M.B., B.C. Camb. | 19 Whiteladies Road, Clifton. |
| WILSON, E. T., M.D. Oxon. | Westol, Cheltenham. |
| *WINDSOR-AUBREY, H. W. | Coronation Road, Bristol. |
| WOODHEAD, G. SIMS, M.D., C.M. Ed. | Pathological Laboratory, New Museum, Cambridge. |
| WOOLLCOMBE, WALTER L. | 16 The Crescent, Plymouth. |

